# Self-Reported Snoring Frequency and Incidence of Cardiovascular Disease: The Circulatory Risk in Communities Study (CIRCS)

**DOI:** 10.2188/jea.JE20110109

**Published:** 2012-07-05

**Authors:** Mako Nagayoshi, Takeshi Tanigawa, Kazumasa Yamagishi, Susumu Sakurai, Akihiko Kitamura, Masahiko Kiyama, Takeo Okada, Kenji Maeda, Tetsuya Ohira, Hironori Imano, Shinichi Sato, Hiroyasu Iso

**Affiliations:** 1 Public Health, Department of Social and Environmental Medicine, Graduate School of Medicine, Osaka University, Suita, Osaka, Japan; 2 Osaka Medical Center for Health Science and Promotion, Osaka, Japan; 3 Department of Public Health, Doctoral Program in Social Medicine, Ehime University Graduate School of Medicine, Toon, Ehime, Japan; 4 Department of Public Health Medicine, Graduate School of Comprehensive Human Sciences, University of Tsukuba, Tsukuba, Ibaraki, Japan; 5 Chiba Prefectural Institute of Public Health, Chiba, Japan

**Keywords:** cardiovascular events, obstructive sleep apnea, population-based study, prospective cohort study

## Abstract

**Background:**

Although associations between snoring and cardiovascular disease have been reported in several prospective studies, there is limited evidence from Asian populations. The objective of this study was to determine if there is an association between self-reported snoring frequency and the incidence of cardiovascular disease in Japanese.

**Methods:**

The subjects were 2350 men and 4163 women aged 40 to 69 years who lived in 3 communities in Japan. All subjects were participants in the Circulatory Risk in Communities Study (CIRCS) and were followed for 6 years. Incidence of cardiovascular disease during the follow-up period comprised events of myocardial infarction, angina pectoris, sudden cardiac death and stroke.

**Results:**

During the 6-year follow-up period, 97 participants (56 men and 41 women) had cardiovascular events. After adjustment for potential confounding factors, self-reported snoring frequency was associated with an increased risk of cardiovascular events among women but not men. The hazard ratios (95% CI) for cardiovascular events were 0.9 (0.4–2.0) for sometimes snoring and 2.5 (1.0–6.1) for everyday snoring in women and 0.7 (0.3–1.3) and 1.0 (0.5–2.1), respectively, in men. Further adjustment for body mass index attenuated the association in women; the respective hazard ratios for cardiovascular events were 0.9 (0.4–1.9) and 2.1 (0.9–5.4).

**Conclusions:**

Self-reported habitual snoring was associated with increased risk of cardiovascular events among Japanese women. Overweight may partly mediate this association.

## INTRODUCTION

Sleep-disordered breathing (SDB) is characterized by repeated episodes of apnea and hypopnea events during sleep.^1^ Recently, SDB was identified as a risk factor for various disorders and diseases such as hypertension,^2^^–^^5^ insulin and glucose abnormalities,^6^ and cardiovascular disease.^7^ Evidence has shown that self-reported snoring is a surrogate marker for SDB.^8^ Associations of snoring and SDB with cardiovascular disease were examined cross-sectionally^9^^,^^10^ in clinical and large-scale epidemiologic studies, most of which clearly showed an independent positive association between the 2 conditions, even after adjustment for potential confounding factors such as age, sex, and body mass index (BMI).^11^

This causal relationship has also been observed in studies of Western populations. As compared with non-snorers, the relative risk of developing cardiovascular disease among habitual snoring American women was 30% higher in the Nurses’ Health Study,^12^ and the risk among habitual and frequent snoring Finish men was 2.1-fold higher.^13^ However, evidence from Asian populations is still very limited, and these results from Western studies cannot simply be extrapolated to Asian populations, because substantial differences exist between Asian and white populations, such as disparities in physique and prevalence of cardiovascular disease subtypes.^14^

In our previous large cross-sectional study,^15^ we reported that the prevalence of snorers among Japanese men was about 70%, despite the low prevalence of obesity. The study revealed that alcohol consumption and cigarette smoking increased snoring risk among Japanese, especially among those who were not overweight. We also reported differences in the distribution of cardiovascular disease subtypes among Japanese versus whites, eg, among Japanese, the proportion of stroke was 2 times higher,^16^ and the proportion of ischemic heart disease was one quarter, compared to the respective values reported in the United Kingdom and United States.^17^ It is thus important to assess the risks of habitual snoring in Asians, because snoring is affected not only by obesity but also other factors, and Asian populations have different distributions of cardiovascular disease subtypes. We therefore examined the risk of cardiovascular events among habitual snorers compared with non-snorers in a large community-based prospective study of Japanese adults.

## METHODS

### Study subjects

Subjects were recruited from participants in the Circulatory Risk in Communities Study (CIRCS, see Appendix), a prospective community-based study of cardiovascular disease in 5 communities across Japan that was launched in 1963.^18^ The subjects of the present sleep study comprised Japanese men and women aged 40 to 69, and none had previously received a diagnosis of SDB. Baseline data on snoring frequency and cardiovascular risk factors were obtained during annual surveys between 2001 and 2005 in the district of Yao City (midwestern suburban community, *n* = 1994), Ikawa (northeastern rural community, *n* = 1446), and between 2000 and 2004 in Kyowa (mideastern rural community, *n* = 3209). We excluded participants with incomplete data on sleep questionnaires (*n* = 82), those with missing data for BMI or other parameters (*n* = 10), and those with a history of ischemic heart disease (*n* = 17) or stroke (*n* = 19). A total of 2350 men and 4163 women were enrolled in the present study. All subjects had the protocol explained in detail and gave their informed consent for participation. The study protocol was approved by the Medical Ethics Committees of the University of Tsukuba.

### Determination of endpoint

Follow-up lasted until the end of 2009 for Ikawa, until the end of 2008 for Yao, and until the end of 2006 for Kyowa. The criteria for ischemic heart disease were modified from those established by the World Health Organization (WHO) Expert Committee.^19^ Definite myocardial infarction was defined as characteristic severe chest pain (lasting for ≥30 min) together with the appearance of new abnormal and persistent Q or QS waves and/or consistent changes in cardiac enzyme activity. Probable myocardial infarction was defined as characteristic chest pain in the absence of electrocardiographic findings or findings related to enzyme activity. Angina pectoris was defined as repeated episodes of chest pain during effort, especially when walking, that usually rapidly disappeared after cessation of effort or use of sublingual nitroglycerin. Sudden cardiac death was defined as death within 1 hour of symptom onset, a witnessed cardiac arrest, or abrupt collapse not preceded by symptoms persisting 1 hour or longer. Stroke was defined as a focal neurological disorder of rapid onset that persisted at least 24 hours or until death. Determination of incident strokes was conducted based on clinical criteria.^20^ A panel of 3 or 4 physician-epidemiologists who were blinded to data from the risk factor surveys made the final diagnoses for these suspected cases of ischemic heart disease and stroke. Cardiovascular disease included events related to ischemic heart disease (definite and probable myocardial infarction, angina pectoris, and sudden cardiac death) and stroke during the follow-up period.

For case ascertainment, histories of cardiovascular events were obtained from annual cardiovascular risk surveys, national insurance claims, ambulance records, reports of local physicians, and public health nurses. To confirm the diagnosis, all living patients were telephoned or visited to obtain a medical history, and their medical records were reviewed. For deaths, we obtained histories from families and reviewed medical records. The protocol has been described in detail elsewhere.^20^^,^^21^

### Risk factor measurements

At the annual cardiovascular surveys, each participant was asked about their snoring frequency. Answer options for the question “Did you snore during the past 3 months?” were almost every day, sometimes, never, and unknown. Information on smoking and drinking habits, menopausal status (for women), and measurements of blood pressure, serum glucose concentration, and physique were obtained according to the CIRCS protocol.^22^ Hypertension was defined as systolic blood pressure of 140 mm Hg or higher, diastolic blood pressure of 90 mm Hg or higher, or antihypertensive treatment; diabetes mellitus was defined as fasting blood glucose of 126 mg/dl or higher, non-fasting blood glucose of 200 mg/dl or higher, or antihyperglycemic treatment; and hypercholesterolemia was defined as serum total cholesterol of 220 mg/dl or higher or treatment with lipid-lowering medication.

### Statistical analyses

Person-years for cardiovascular events were calculated as the sum of individual follow-up time until cardiovascular event, emigration, or the end of the follow-up period. Age- and community-adjusted and multivariable-adjusted hazard ratios (HRs) and 95% CIs for cardiovascular events were calculated according to baseline snoring frequency by using the Cox proportional hazards model. The interaction of snoring with sex in relation to cardiovascular events was tested using their cross-product terms.

Confounding variables included age (continuous), BMI (continuous), alcohol consumption (never, ex-, <23 g and ≥23 g ethanol per day), cigarette smoking (never, ex-, <20 and ≥20 cigarettes per day), community (categorical), and menopausal status (for women; yes, no). To confirm the hypothesis that snoring induces cardiovascular events by increasing the risk of hypertension and metabolic disorders, we further adjusted for systolic blood pressure (continuous), use of antihypertensive medication (dichotomous), diabetes mellitus (dichotomous), and hypercholesterolemia (dichotomous). All statistical analyses were performed using SAS version 9.1 software (SAS Institute Inc., Cary, NC, USA). All statistical tests were 2-tailed, and *P* values less than 0.05 were regarded as statistically significant.

## RESULTS

### Demographic characteristics at baseline

The mean (± SD) age of participants was 56.3 ± 8.3 years, mean BMI was 23.5 ± 3.2 kg/m^2^, mean systolic blood pressure was 132.3 ± 18.6 mm Hg, and mean diastolic blood pressure was 80.4 ± 11.1 mm Hg.

Analysis revealed that 42.5% of participants had hypertension, 17.5% were using antihypertensive medication, 5.9% had diabetes mellitus, 44.7% had hypercholesterolemia, 37.6% were current drinkers, 21.1% were current smokers, and 69.1% of women were post-menopausal. Baseline demographic characteristics, by sex, are shown in Table 1.

**Table 1.** Sex-specific mean values (SD) and prevalence of selected cardiovascular risk characteristics among 2350 men and 4163 women aged 40–69 years

	Men	Women		*P* value
	*n* = 2350	*n* = 4163		
Age (years)	57.5 (8.2)	55.6 (8.3)		<0.001
Body mass index (kg/m^2^)	23.9 (3.0)	23.3 (3.3)		<0.001
Systolic blood pressure ​ (mm Hg)	135.1 (17.6)	130.7 (18.9)		<0.001
Diastolic blood pressure ​ (mm Hg)	83.3 (10.9)	78.7 (10.8)		<0.001
Antihypertensive use (%)	19.2	16.5		0.005
Hypertension (%)^a^	51.1	37.6		<0.001
Diabetes mellitus (%)^b^	9.1	4.1		<0.001
Hypercholesterolemia (%)^c^	34.5	50.5		0.28
Alcohol consumption (%)				
never	19.6	78.5	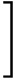	<0.001
ex-drinker	7.3	4.0		
<23 g ethanol per day	22.6	15.1		
≥23 g ethanol per day	50.6	2.4		
Cigarette smoking (%)				
never	17.0	90.5	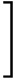	<0.001
ex-smoker	35.4	3.3		
<20 cigarettes per day	13.2	4.4		
≥20 cigarettes per day	34.3	1.8		
Menopause (%)	—	69.1		—

^a^Hypertension was defined as blood pressure ≥140/90 mm Hg or current treatment.

^b^Diabetes mellitus was defined as fasting blood glucose ≥126 mg/dl, non-fasting blood glucose ≥200 mg/dl, or current treatment.

^c^Hypercholesterolemia was defined as total cholesterol ≥220 mg/dl or current treatment.

### Prevalence of snoring and its correlates

The distribution of snoring frequency was as follows: 14.0% (23.9% in men and 8.5% in women) reported snoring almost every day, 46.7% (48.7% in men and 45.6% in women) reported snoring sometimes, 28.9% (20.7% in men and 33.6% in women) reported never snoring, and 10.3% (6.8% in men and 12.3% in women) reported that their snoring frequency as unknown. Sex-specific, age-adjusted characteristics classified according to snoring frequency are shown in Table 2. The mean values and proportions of baseline risk characteristics tended to be higher with increasing snoring frequency, except for mean age in men and women and prevalence of diabetes mellitus in men. As compared with women who reported a snoring frequency, those who answered “unknown” were 2.6 years older (*P* < 0.001), 0.5 points lower in mean BMI (*P* = 0.001), and 4.3% higher in the mean proportion of current smokers (*P* < 0.001) in women. There was no significant difference among men.

**Table 2.** Sex-specific, age-adjusted, mean values (standard error) and prevalence of selected cardiovascular risk characteristics according to snoring frequency among 2350 men and 4163 women aged 40–69 years

Snoring frequency	Men				Women			
	
	Never	Sometimes	Daily	Unknown	Never	Sometimes	Daily	Unknown
	*n* = 486	*n* = 1144	*n* = 561	*n* = 159	*n* = 1399	*n* = 1900	*n* = 352	*n* = 512
Age (years)^a^	59.0 (0.4)	57.1 (0.2)	56.8 (0.3)	57.9 (0.6)	55.1 (0.2)	55.2 (0.2)	55.9 (0.4)	57.9 (0.4)
Body mass index (kg/m^2^)	23.1 (0.1)	23.8 (0.1)	24.9 (0.1)	23.6 (0.2)	22.6 (0.1)	23.6 (0.1)	24.9 (0.2)	22.8 (0.1)
Systolic blood pressure (mm Hg)	133.4 (0.8)	135.1 (0.5)	136.5 (0.7)	135.0 (1.3)	128.3 (0.5)	132.4 (0.4)	133.5 (0.9)	129.0 (0.8)
Diastolic blood pressure (mm Hg)	81.9 (0.5)	83.0 (0.3)	85.3 (0.5)	83.2 (0.9)	77.0 (0.3)	79.6 (0.2)	80.8 (0.6)	78.6 (0.5)
Antihypertensive use (%)	18.6	17.6	22.1	22.8	13.3	18.1	22.3	15.0
Hypertension (%)^b^	48.3	49.3	56.8	52.2	31.5	41.4	46.0	34.8
Diabetes mellitus (%)^c^	9.2	9.2	8.8	9.3	3.4	4.3	6.2	3.8
Hypercholesterolemia (%)^d^	30.2	35.6	37.4	28.9	46.7	51.8	55.4	52.7
Cigarette smoking (%)								
never	22.4	15.6	15.0	18.2	92.5	91.1	87.4	84.8
ex-smoker	34.5	35.8	36.1	33.7	2.6	3.2	4.3	5.2
<20 cigarettes per day	13.6	14.1	10.9	13.7	3.9	4.2	4.6	6.2
≥20 cigarettes per day	29.5	34.6	38.1	34.3	1.0	1.6	3.7	3.8
Alcohol consumption (%)								
never	23.2	19.0	17.4	20.2	82.5	77.8	72.5	74.8
ex-drinker	10.5	6.2	6.1	9.9	3.1	3.8	6.6	5.1
<23 g ethanol per day	21.9	24.2	21.0	18.4	13.2	16.1	16.1	16.1
≥23 g ethanol per day	44.4	50.6	55.6	51.5	1.2	2.3	4.9	4.0
Menopause (%)	—	—	—	—	67.1	70.0	71.5	69.2

^a^Age was not included in the adjustment variables.

^b^Hypertension was defined as blood pressure ≥140/90 mm Hg or current treatment.

^c^Diabetes mellitus was defined as fasting blood glucose ≥126 mg/dl, non-fasting blood glucose ≥200 mg/dl, or current treatment.

^d^Hypercholesterolemia was defined as total cholesterol ≥220 mg/dl or current treatment.

### Incidence of cardiovascular events

During the 6-year median follow-up duration, 97 participants (56 men and 41 women) experienced cardiovascular events, including 30 (22 in men and 8 in women) incident cases of ischemic heart disease and 67 (34 in men and 33 in women) strokes. The numbers of ischemic heart disease and stroke events, according to snoring frequency and sex, are shown in Table 3.

**Table 3.** Sex-specific age- and community-adjusted and multivariable-adjusted hazard ratios (95% CI) for incidence of cardiovascular events according to snoring frequency

Snoring frequency	Men				Women			
	
	Never	Sometimes	Daily	Unknown	Never	Sometimes	Daily	Unknown
Person-years	2792	7014	3427	951	8462	11 715	2119	3268
Subjects (*n*)	486	1144	561	159	1399	1900	352	512
Incident cardiovascular event (*n*)	15	22	16	3	13	16	8	4
Incident ischemic heart disease (*n*)	7	10	7	2	1	4	2	1
Incident stroke (*n*)	8	12	9	1	12	12	6	3
Age- and community-adjusted HR ​ (95% CI)	(Reference)	0.7 (0.4–1.4)	1.1 (0.5–2.2)	0.7 (0.2–2.3)	(Reference)	0.9 (0.5–2.0)	2.6 (1.1–6.3)	0.8 (0.2–2.4)
Model 1 HR (95% CI)^a^	(Reference)	0.7 (0.3–1.3)	1.0 (0.5–2.1)	0.7 (0.2–2.3)	(Reference)	0.9 (0.4–2.0)	2.5 (1.0–6.1)	0.8 (0.2–2.4)
Model 2 HR (95% CI)^b^	(Reference)	0.6 (0.3–1.3)	1.0 (0.5–2.0)	0.6 (0.2–2.3)	(Reference)	0.9 (0.4–1.9)	2.1 (0.9–5.4)	0.8 (0.2–2.4)
Model 3 HR (95% CI)^c^	(Reference)	0.6 (0.3–1.2)	0.9 (0.4–1.9)	0.6 (0.2–2.1)	(Reference)	0.8 (0.4–1.7)	1.9 (0.8–4.9)	0.7 (0.2–2.2)

HR: hazard ratio; CI: confidence interval.

^a^Model 1 was adjusted for age, alcohol consumption, cigarette smoking, community, and, for women, menopausal status at baseline.

^b^Model 2 was adjusted for factors in Model 1 plus body mass index.

^c^Model 3 was adjusted for factors in Model 2 plus systolic blood pressure, antihypertensive medication use, diabetes mellitus, and hypercholesterolemia.

### Association of snoring with cardiovascular events

As compared with never snorers, the age- and community-adjusted HR of ischemic heart disease and stroke combined was higher among female but not male everyday snorers; the HRs (95% CI) for cardiovascular events were 0.9 (0.5–2.0) for sometimes snoring and 2.6 (1.1–6.3) for everyday snoring in women and 0.7 (0.4–1.4) and 1.1 (0.5–2.2), respectively, in men (Table 3). These associations in men did not vary by age group (40–59 and 60–69 years), smoking status (current and non-current), or drinking status (current and non-current) (data not shown in table). After adjustment for age, community, and other confounding variables, the association between self-reported snoring frequency and cardiovascular events was unchanged in women: the HRs (95% CI) for cardiovascular events were 0.9 (0.4–2.0) for sometimes snoring and 2.5 (1.0–6.1) for everyday snoring in women (Table 3, Model 1). Further adjustment for BMI attenuated the association in women: the respective HRs for cardiovascular events were 0.9 (0.4–1.9) and 2.1 (0.9–5.4; Table 3, Model 2). To confirm the hypothesis that snoring induces cardiovascular events by increasing the risk of hypertension and metabolic disorders, we further adjusted for systolic blood pressure, antihypertensive medication use, diabetes mellitus and hypercholesterolemia. The respective HRs (95% CI) for cardiovascular events were 0.8 (0.4–1.7) and 1.9 (0.8–4.9; Table 3, Model 3). The association between everyday snoring and risk of cardiovascular events was not significantly modified by sex (*P* for interaction = 0.12). The risk of cardiovascular events associated with unknown snoring was not increased and was similar to the risk associated with sometimes snoring in both men and women.

## DISCUSSION

In the present prospective study, snoring frequency was associated with an increased incidence of cardiovascular events among community-dwelling middle-aged Japanese women. This association was independent of age and other confounding factors. As compared with never snorers, ‘everyday snoring’ women had a 2.5-fold higher risk of cardiovascular events during 6 years of follow-up. The association of everyday snoring with cardiovascular events was attenuated after adjustment for BMI and after further adjustment for systolic blood pressure, antihypertensive medication use, diabetes mellitus, and hypercholesterolemia. This suggested that overweight partly mediated the association and that hypertension and metabolic abnormalities partly caused by snoring contribute to the risk of cardiovascular events in women who snore every day. This is the first study to show a relationship between habitual snoring and risk of cardiovascular events among a population in Asia, which has a low prevalence of obesity.

The biological mechanisms that link habitual snoring to the development of cardiovascular disease remain to be fully elucidated, but a number of mechanisms have been proposed. Habitual snoring is often accompanied by sleep apnea or hypopnea. Repetitive episodes of intermittent complete and partial airway collapse during sleep result in hypoxemia, hypercapnia, changes in intrathoracic pressure, and repeated arousal from sleep. Episodes of snoring and apneic events can cause acute hemodynamic changes^11^ (such as increased cardiac output, enhanced cardiac arrhythmia, patent foramen ovale appearance,^23^ increased intracranial pressure, and decreased cerebral blood flow),^24^ increased platelet aggregation^25^ and fibrinogen concentrations,^26^ and decreased fibrinolysis, which directly affect the cardiovascular system. Abnormal metabolic conditions such as hypertension, diabetes mellitus, and hypercholesterolemia may also increase the risk of cardiovascular disease via elevation of sympathetic activation,^27^^,^^28^ oxidative stress,^29^ activation of the hypothalamic-pituitary-adrenal axis due to sleep fragmentation,^30^^,^^31^ and endothelial dysfunction.^32^

In the present study, habitual snorers were more likely to be overweight, hypertensive, and diabetic than non-snorers, and the association between snoring frequency and the risk of cardiovascular events was attenuated when we further adjusted for these factors. This suggests that overweight partly mediates the association and that habitual snoring increases the risk of cardiovascular events partly through increasing the risk of hypertension and metabolic disorders.

The present results are consistent with those from studies of Western women^12^ but not men^13^; however, this study is the first to note an independent association in a population with a different distribution of cardiovascular disease subtypes and a low prevalence of obesity. Among Japanese, 70% of cardiovascular events are strokes—whereas in Western countries ischemic heart disease is the largest cause of such events—and the risk factors for cardiovascular events among populations with low obesity are hypertension and metabolic abnormalities rather than overweight.^33^

In contrast to the significant association of habitual snoring with cardiovascular events in women, no such association was observed in men. Large population-based prospective studies of middle-aged men,^13^ women,^12^ and a population of men and women aged 20 years or older^34^ have reported positive associations between habitual snoring and cardiovascular events. However, no study has reported a sex difference in the association. Recent reports from the Sleep Heart Health Study (a large population-based study of American residents aged 40 or older) have noted sex differences in the association between SDB, as defined by the apnea-hypopnea index (AHI), and the risk of coronary heart disease, heart failure, and stroke.^35^^,^^36^ The multivariable HRs associated with a 10-unit increase in AHI were 1.1 (1.0–1.3) for incident heart failure in men and 1.1 (1.0–1.2) for incident coronary heart disease in men aged 70 years or younger, whereas no such associations were observed in women.^35^ Similarly, the multivariable HR for ischemic stroke incidence was 2.9 (1.1–7.4) in men and 1.2 (0.7–2.2) in women for the highest (>19) as compared with the lowest (≤4) AHI quartiles.^36^ The reasons for the present lack of association between habitual snoring and risk of cardiovascular events in men are unknown. We found no association in men when the analysis was stratified by age, smoking, or drinking status. Further research is necessary to elucidate this sex difference.

The strengths of the present study include the use of systematic surveillance of cardiovascular events and complete data collection on incident stroke and ischemic heart disease, including sudden cardiac death. Our large population-based prospective cohort study enabled us to examine sex-specific associations between snoring frequency and risk of cardiovascular events and provides the first evidence of a positive association between these conditions in an Asian population.

The limitations of the present study are as follows: first, our data on snoring frequency were obtained from a self-reported questionnaire, so lack of awareness of snoring or the absence of a sleep partner may have resulted in misclassification. However, in our simultaneous subsample study (1564 men and 2806 women aged 40–69 years) using a 3% oxygen desaturation index (ODI) measured by pulse oxymetry (PULSOX-3Si; Minolta, Osaka, Japan) during 1 night of sleep at a participants’ homes, we found that the proportion of SDB (ODI ≥5 events/hours) was 22% in never snorers, 36% in sometime snorers, and 50% in everyday snorers among men. The respective proportions for women were 9%, 19%, and 34%. Thus, self-reported snoring seemed to be reliable. Second, data on sleep duration were not obtained in this study. According to a recent meta-analysis, both short (≤5–6 hours per night in most studies) and long sleep duration (≥8–9 hours per night in most studies) were associated with increased risks of coronary heart disease and stroke.^37^ Further studies of the effects of sleep quality and quantity on the risk of cardiovascular disease will be necessary to confirm an effect of habitual snoring.

In summary, the present large cohort study showed that habitual snoring was associated with an increased risk of cardiovascular events among community-dwelling middle-aged Japanese women and that overweight, snoring-related hypertension, and metabolic disorders may partly mediate the association. The present study provides epidemiologic evidence for physicians and other health professionals that habitual snoring should be considered in the prevention of cardiovascular disease among middle-aged Japanese.

## APPENDIX

### CIRCS investigators

The Circulatory Risk in Communities Study (CIRCS) is a collaborative study managed by the Osaka Medical Center for Health Science and Promotion, Osaka University, University of Tsukuba, and Ehime University. CIRCS investigators who contributed to this study are as follows: Hiroyasu Iso, Tetsuya Ohira, Hironori Imano, Renzhe Cui, Ai Ikeda, Hiroyuki Noda, Satoyo Ikehara, Isao Muraki, and Kotatsu Maruyama, Osaka University, Suita; Tomoko Sankai and Kazumasa Yamagishi, Mitsumasa Umesawa, Choy-Lye Chei, Kimiko Yokota, and Minako Tabata, University of Tsukuba, Tsukuba; Masamitsu Konishi, Yoshinori Ishikawa, Masakazu Nakamura, Akihiko Kitamura, Masahiko Kiyama, Takeo Okada, Kenji Maeda, Masatoshi Ido, Masakazu Nakamura, Takashi Shimamoto, Minoru Iida, and Yoshio Komachi, Osaka Medical Center for Health Science and Promotion, Osaka; Shinichi Sato, Chiba Prefectural Institute of Public Health, Chiba; Takeshi Tanigawa, Isao Saito, Susumu Sakurai, and Shinichi Hitsumoto Ehime University, Toon; Masayuki Yao, Ranryoen Hospital, Ibaraki.
